# Genetic Population Structure in the Antarctic Benthos: Insights from the Widespread Amphipod, *Orchomenella franklini*

**DOI:** 10.1371/journal.pone.0034363

**Published:** 2012-03-27

**Authors:** Helena Phoenix Baird, Karen Joy Miller, Jonathan Sean Stark

**Affiliations:** 1 Institute for Marine and Antarctic Studies, University of Tasmania, Hobart, Tasmania, Australia; 2 Terrestrial and Nearshore Ecosystems Theme, Australian Antarctic Division, Kingston, Tasmania, Australia; Macquarie University, Australia

## Abstract

Currently there is very limited understanding of genetic population structure in the Antarctic benthos. We conducted one of the first studies of microsatellite variation in an Antarctic benthic invertebrate, using the ubiquitous amphipod *Orchomenella franklini* (Walker, 1903). Seven microsatellite loci were used to assess genetic structure on three spatial scales: sites (100 s of metres), locations (1–10 kilometres) and regions (1000 s of kilometres) sampled in East Antarctica at Casey and Davis stations. Considerable genetic diversity was revealed, which varied between the two regions and also between polluted and unpolluted sites. Genetic differentiation among all populations was highly significant (*F*_ST_ = 0.086, *R*_ST_ = 0.139, p<0.001) consistent with the brooding mode of development in *O. franklini*. Hierarchical AMOVA revealed that the majority of the genetic subdivision occurred across the largest geographical scale, with *N_e_m*≈1 suggesting insufficient gene flow to prevent independent evolution of the two regions, i.e., Casey and Davis are effectively isolated. Isolation by distance was detected at smaller scales and indicates that gene flow in *O. franklini* occurs primarily through stepping-stone dispersal. Three of the microsatellite loci showed signs of selection, providing evidence that localised adaptation may occur within the Antarctic benthos. These results provide insights into processes of speciation in Antarctic brooders, and will help inform the design of spatial management initiatives recently endorsed for the Antarctic benthos.

## Introduction

Gene flow – or genetically effective migration – is one of the most important factors governing the evolution of species [1]–[3]. Gene flow can dampen localised adaptation yet spread advantageous alleles for the cohesive evolution of the species (e.g. in the face of climate change), whereas the absence of gene flow can lead to population divergence, and ultimately speciation [4]–[6]. Anthropogenic impacts have the potential to disrupt gene flow and alter genetic diversity of natural populations (e.g. [7]), thereby affecting the evolutionary potential of species. Understanding the genetic structure of populations will therefore shed light on how species may respond to these impacts. Moreover, genetic studies can help optimise the design of conservation efforts to preserve genetic diversity, in order to help ensure the long-term adaptability and persistence of species [8], [9].

Marine fauna are currently threatened by a plethora of human activities including fisheries harvest, habitat destruction, localised pollution, introduced species and climate change (see [10], [11]). Initiatives such as Marine Protected Areas (MPAs) are one of the most important tools for the conservation of marine populations, the success of which relies on ‘spillover’ of individuals from protected areas to replenish outside populations [12]–[14]. Estimates of genetic connectivity thus help determine the optimal size and placement of MPAs to achieve this desired broad-scale flux [15], [16]. There is strong evidence of a correlation between the dispersal capacity inferred by a species' pelagic larval phase and the genetic connectivity of populations (and subsequent potential for MPA connectivity), however exceptions are common, and patterns of marine genetic structure are far from predictable (reviewed in [17]–[19]). Furthermore, local adaptation has been increasingly emphasised for its role in structuring marine populations (e.g. [20]–[22]). Local adaptation may be particularly important in the face of localised marine pollution, which can alter allele frequencies or genetic diversity in exposed populations (e.g. [23]–[25]), in turn affecting speciation processes and ultimately, species fitness [26].

The Antarctic benthos represents one of the most isolated marine ecosystems on the planet, with particularly unique fauna (reviewed in [27], [28]) that are considered vulnerable to future environmental change [29]. Intraspecific genetic structure in Antarctic benthic organisms is poorly understood, and the need for genetic research has been highlighted [30]–[33]. This is particularly pertinent given a recent endorsement by the Convention for the Conservation of Antarctic Marine Living Resources (CCAMLR) to establish a network of MPAs in Antarctica [34]. Determining the location and size of these MPAs is still in progress and is hindered by a lack of existing baseline knowledge on Antarctic marine fauna [35]. Improving our understanding of benthic gene flow and genetic diversity in Antarctica will not only help inform the design of these MPAs, but will also shed light on the high rates of speciation prevalent in Antarctic benthos [30], [32], [36]. Furthermore, studying these microevolutionary processes will help predict the potential for Antarctic organisms to adapt to existing threats, such as local pollution surrounding human settlements [37], [38], and broad-scale climate change [39].

Studies of gene flow in Antarctic benthic fauna have focused primarily on large-scale connectivity over major hydrographic features such as the Polar Front, or abyssal depths between islands (e.g. [40]–[42]). Commonly, these studies have revealed highly distinct genetic lineages assumed to represent cryptic species (see [43] for recent summary). This partly explains why truly *intra*specific genetic patterns remain much less explored. What has emerged from the limited population-level studies is that the unique hydrography of Antarctica may have an important influence on genetic structure. For instance, local circulation patterns are believed to play a role in isolating populations of species that display surprisingly fine-scale (<20 km) genetic subdivision, despite possessing pelagic larvae for dispersal (Notothenioidei: [44]; Bivalvia: [45]). Other unique mechanisms such as iceberg scouring and historical glaciation have been implicated when populations from different locations exhibit markedly different levels of genetic diversity (e.g. Amphipoda: [46]; Isopoda: [47], Ascidiacea: [48]; Pycnogonida: [49]).

Brooding benthic organisms are particularly interesting candidates in which to address questions of gene flow and speciation in Antarctica, as their lack of a pelagic dispersal phase should lead to high genetic structuring of populations [50], [51]. In Antarctica, brooding taxa are highly speciose and largely endemic, with several groups that have undergone intense radiation [52]–[55]. One such group is the amphipods, which are remarkably abundant crustaceans that occupy a wide range of ecological niches and play a significant role in Antarctic trophic exchanges [50], [56]–[58]. We chose to study the ubiquitous amphipod *Orchomenella franklini* (Walker, 1903) to address the current paucity of knowledge on intraspecific genetic structure in Antarctic benthos. *O. franklini* often dominates Antarctic shallow water communities [59]–[62], and its presence in polluted bays adjacent to Antarctic research stations allowed us to investigate potential effects of contamination on genetic diversity.

We used microsatellite markers to investigate genetic variation in *O. franklini*. Microsatellites are tandem repeats of typically one to six nucleotides that mutate rapidly and are found at high frequency in most eukaryotic genomes [63], [64]. The high variability of microsatellites enables resolution of genetic structure over fine (<100 km) spatial scales [32], [63]–[67], which in Antarctica remain the least understood [32], [68]. To our knowledge just a single study to date has used microsatellites to explore gene flow in an Antarctic benthic invertebrate, and this focused on large-scale migration between islands [48]. Thus we present the first known Antarctic study of microsatellite variation in a benthic invertebrate over small (100 s of metres), moderate (10 s of kilometres), and large (1000 s of kilometres) spatial scales.

## Materials and Methods

### Sampling

**Figure 1:**
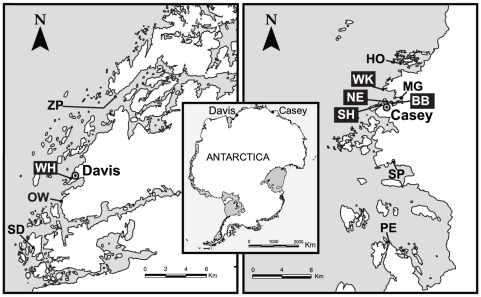
Maps showing the regions and locations sampled for *Orchomenella franklini*. Map of Antarctica shows the relative position of Casey and Davis; close-up maps of each region show the locations sampled (dark boxes indicate polluted locations). Locations are Zappet (ZP), Wharf (WH), Old Wallow (OW), Sorsdal (SD), Honkala (HO), Wilkes (WK), McGrady (MG), Newcombe (NE), Brown (BB), Shannon (SH), Sparkes (SP) and Peterson (PE). Within each location one to four replicate sites were sampled at a depth <10 m (see Table S1 for further region, location and site details). Images derived from base maps courtesy of the Australian Antarctic Data Centre.

Samples of *Orchomenella franklini* were collected from two geographical regions, adjacent to the Australian research stations Casey (66°S, 110°E) and Davis (68°S, 78°′E), in East Antarctica. Casey and Davis are separated by approximately 1400 km (Fig. 1), and were sampled during the summer months of 2009 and 2010 respectively. Within the Casey region samples were collected at eight locations (∼1–30 km apart; Fig. 1). Within the Davis region samples were collected at four locations (∼3–20 km apart; Fig. 1). Locations were classified as either polluted or unpolluted (Fig. 1, Table S1) based on proximity to known contaminated areas [69]–[71], and knowledge of the extent of dispersion of these contaminants [71], [72], which include hydrocarbons, heavy metals and faecal sterols. Within each location two to four sites were sampled 100 m apart, except in two instances where only a single site was accessible due to local ice conditions (Table S1). At each site a van-veen grab was used to take a small (<1 m^3^) sample of the benthic sediment from no more than 10 m water depth. This sediment was sieved on a 0.5 mm mesh and retained fauna were sorted under a dissecting microscope. All identified *O. franklini* specimens were removed and stored at 4°C in vials of 80% ethanol (see Table S1 for final sample sizes). All necessary permits were obtained for the described field studies from the Commonwealth of Australia under the Antarctic Marine Living Resources Act 1981. Collections from Casey were made under permit AMLR 08-09-3051 and collections from Davis were made under permit AMLR 09-10-3051; voucher specimens are held at the Australian Antarctic Division.

### Development of microsatellite loci

A genomic library of *O. franklini* was made by ecogenics Gmbh (Zurich, Switzerland) based on a pooled sample of DNA from 15 individual amphipods. SNX forward and reverse linkers were ligated onto size-selected DNA following the procedure of [73] and these were enriched for (TAC)_10_, (AAC)_10_, (GT)_13_, (CT)_13_ and (ACAG)_7_ oligonucleotide repeats by magnetic bead selection [74], [75]. The enriched library was cloned and 1406 recombinant colonies screened for the presence of microsatellites by hybridisation. DNA inserts from 230 positive clones were subsequently sequenced and primers were designed for 23 microsatellite loci and tested for polymorphism. Seven loci (Table 1) were considered suitable for population-level analysis.

**Table 1:** Details for the seven microsatellite loci amplified in *Orchomenella franklini*.

Multiplex	PCR thermal protocol	Locus	Primer sequence (5′-3′)	Flourescent dye	Repeat motif	Total no. of alleles	Allele size range (bp)
**A**	15 min at 95°C	*Orcfra4*	F: AGCAGTCCCTAACAGAAATGG	D2	(AAC)_7_	9	97–116
	[30 s at 94°C, 90 s at 58.5°C, 60 s at 72°C] x31		R: GGCGCTCCAATAAGTTCTTC				
	30 min at 72°C	*Orcfra5*	F: GTGGGGGCTACGGTAGAAAC	D3	(CAA)_7_	6	138–159
			R: TTGTTTGTATTGCTCTTGTAACTATTG				
**B**	15 min at 95°C	*Orcfra3*	F: AAACACAGCCCCAGTTGATG	D2	(CAA)_9_	8	228–249
	[30 s at 94°C, 90 s at 61.5°C, 60 s at 72°C] x31		R: TACCATCCCAGGACCACAAG				
	30 min at 72°C	*Orcfra13*	F: AGATGCTGTATTATACTCGTGCTG	D3	(TGT)_6_	7	113–128
			R: CGATCTGCAACATAAACAACAAC				
		*Orcfra26*	F: CGAGCCTGTGCACTCCTAC	D4	(CA)_4_GA(CA)_7_	8	157–173
			R: CGGTGGATAGTTGTTCATGC				
**C**	15 min at 95°C	*Orcfra6*	F: TGTAGACATCACTGCTGGTTAGG	NED	(CTA)_6_	18	86–102
	[30 s at 94°C, 30 s at 55°C, 60 s at 72°C] x40		R: TCGTTTTGCATCAAGACCAC				
	10 min at 72°C	*Orcfra12*	F: CCGGGGTTCTATGAATTACC	FAM	(CTAC)_21_	24	197–237
			R: AGCGCTAAGTGGTGATGAAG				

PCR thermal protocols and corresponding primer details are given for each multiplex reaction. Fluorescent dyes were used to label the forward primers. The number of alleles provided is the total observed across all individuals.

### Microsatellite genotyping

Whole specimens of *O. franklini* were used for DNA extractions due to small body size (2–8 mm). DNA was extracted using the DNeasy Blood and Tissue Kit (QIAGEN) according to the manufacturer's instructions, with elution volume decreased to 120 µl to maximise DNA concentration. Microsatellite loci were amplified in three multiplex polymerase chain reactions (PCRs) (Table 1). For multiplex reactions A and B, 20 µl reactions using the QIAGEN Multiplex PCR kit were used, with a final concentration of 1× Qiagen Multiplex PCR Master Mix (provides 3 mM MgCl and one unit HotStar Taq DNA Polymerase), 0.2 µM each primer (forward primers were fluorescently labeled), and approximately 100 ng template DNA. Capillary separation of amplified fragments occurred on an automated sequencer (CEQ 8000, Beckman Coulter), and alleles were scored (according to PCR fragment size) with CEQ 8000 Genetic Analysis System software version 8.0. Multiplex C was amplified at the Australian Genome Research Facility, with capillary separation occurring on an Applied Biosystems 3730 DNA Analyser and alleles scored using Applied Biosystems GeneMapper software version 3.1. All fragment data were visually checked for allele scoring errors and stutter. Micro-checker 2.2.3 [76] was used to check data for the presence of null alleles. Loci were tested for linkage disequilibrium in Genepop 4.0.10 [77] with the critical level p<0.05 adjusted for multiple comparisons, using the sequential Bonferroni procedure [78].

### Genetic diversity of populations

Measures of genetic diversity, including observed heterozygosity (*H_O_*), unbiased expected heterozygosity (*H_E_*) and allelic richness (standardised for sample size: *A_R_*) at each locus were calculated in FSTAT 2.9.3 [79]. To determine whether diversity measures differed between Casey and Davis, and among polluted and unpolluted sites, permutation tests were performed on *H_O_*, *H_S_* and *A_R_* in FSTAT. The number of private alleles (*P_A_*) at each site was determined using Genalex 6.41 [80]. There is evidence that the occurrence of private alleles most closely follows a Poisson distribution [81], so we used a Poisson generalised linear model to assess the effect of region and pollution on *P_A_*, carried out in R 2.12.2 [82].

Exact tests (using default Markov chain parameters, performed in Genepop) were used to test for departures from Hardy-Weinberg Equilibrium, with Wright's fixation index (*F*_IS_) for each locus-site combination used to determine the nature of those departures (where *F*_IS_<0 indicates heterozygote excess and *F*_IS_>0 indicates heterozygote deficits). Significance levels were adjusted for multiple comparisons using the sequential Bonferroni procedure [78].

Cryptic species are common in Antarctic benthic invertebrate fauna [51], in part because many taxa are poorly studied. To check for the presence of cryptic species within *O. franklini*, Principal Coordinate Analysis (PCoA in Genalex) was used to examine the complete multilocus data set for evidence of distinct genetic groups [83], [84].

### Population differentiation

We calculated Weir and Cockerham's *F*_ST_ estimates [85] to examine genetic differentiation among all sites. Since *F*_ST_ assumes an infinite allele model of mutation (IAM), we also calculated *R*_ST_ which assumes a stepwise mutation model (SMM). Currently there is no consensus over which model is more appropriate for microsatellite data, so the conservative approach is to calculate both [86]. *F*_ST_ and *R*_ST_ were calculated in Arlequin 3.11 [87], using 50000 permutations to assess significance. The high variability of microsatellites can result in depressed estimates of *F*_ST_, therefore we also calculated ‘
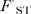
’ (a standardised measurement which accounts for high within-population variation), using RECODEDATA 0.1 [88]. Matrices of the pairwise differentiation (as both *F*_ST_ and *R*_ST_) between all sites were generated in Genepop.

The partitioning of genetic variation among regions, locations nested within regions, sites nested within locations and individuals nested within sites was determined using a four-level hierarchical analysis of molecular variance (AMOVA). AMOVA was performed using Hierfstat [89], which calculates variance components and *F*-statistics [85] for each hierarchical level according to [90]. Departures from values expected under panmixis (i.e. *F*_ST_ = 0) at each hierarchical level were determined with 10,000 permutations of the data. We estimated the migration occurring between Casey and Davis as *N_e_m* = 1/4(1/*F*_ST_−1) [91], using the *F*_ST_ among regions generated from hierarchical AMOVA. *F*_ST_ varied considerably among loci (see results), so we tested for evidence of selection at each of the seven loci using Lositan [92]. Confidence intervals (99%) for neutral loci were determined using 20,000 simulations and the recommended ‘neutral mean *F*_ST_’ option [93].

Coastal marine populations are unlikely to disperse according to the island model [94] so we tested for evidence of isolation by distance (which indicates a stepping-stone mode of dispersal: [95]) among *O. franklini* populations within the Casey and Davis regions. We examined the relationship between geographic distance (as the natural logarithm of the shortest water-based route) and genetic differentiation (as linearised *F*_ST_; i.e. *F*_ST_/(1−*F*_ST_)) between all sites within each region, using Mantel tests implemented in Genepop. The shortest water-based route was an estimation of the direct linear distance (or combination of linear vectors) that an amphipod could feasibly travel between sites (there was insufficient data on ice cover or currents to allow for a more biologically-relevant estimate). Mantel tests were also performed assuming the SMM (i.e. using *R*_ST_/(1−*R*_ST_) for genetic differentiation estimates). Data were permuted 10,000 times to determine significance.

## Results

### Genetic diversity of populations

A total of 718 *Orchomenella franklini* specimens were genotyped for the seven microsatellite loci (448 from Casey, 270 from Davis: Table S1). None of the loci showed evidence of linkage disequilibrium (p>0.05 for all pairwise comparisons). The number of alleles observed at each locus ranged from 6 to 24 (Table 1). Average allelic richness, observed heterozygosity and expected heterozygosity were all significantly higher at Davis (*A_R_* = 4.46, *H_O_* = 0.495, *H_E_* = 0.574), compared to Casey (*A_R_* = 3.62, *H_O_* = 0.428, *H_E_* = 0.447; p<0.001; Table 2). None of these diversity measures were found to differ significantly among polluted and unpolluted sites within either of the regions.

**Table 2:** Genetic diversity of *Orchomenella franklini* populations from Casey and Davis.

	Casey			Davis		
	*A_R_*	*H_E_*	*H_O_*	*A_R_*	*H_E_*	*H_O_*
*Orcfra3*	4.655	0.730	0.709	4.768	0.691	0.663
*Orcfra4*	1.848	0.118	0.089	3.705	0.630	0.230
*Orcfra5*	1.547	0.081	0.053	2.404	0.197	0.128
*Orcfra6*	6.009	0.814	0.795	5.409	0.645	0.584
*Orcfra12*	6.975	0.812	0.766	9.287	0.895	0.900
*Orcfra13*	2.963	0.533	0.542	3.066	0.522	0.541
*Orcfra26*	1.338	0.040	0.041	2.567	0.438	0.422
Overall	3.619	0.447	0.428	4.458	0.574	0.495

Allelic richness (*A_R_*), expected heterozygosity (*H_E_*), and observed heterozygosity (*H_o_*) are given for each microsatellite locus, and averaged over all loci.

A total of 16 private alleles were observed across the entire dataset (Table S2). Standard diagnostics revealed that the Poisson generalised linear model was an appropriate model for the number of private alleles per population (*P_A_*), with no evidence of overdispersion. The model revealed strong evidence that *P_A_* was significantly greater at Davis than at Casey (p = 0.002), and also significantly greater at unpolluted sites compared to polluted sites (p = 0.005; Table S2). There was no evidence of a significant interaction between the two factors (p = 0.307), and average sample size was almost identical for unpolluted and polluted populations, thus could be disregarded as a potential confounding factor.

Most populations were in Hardy-Weinberg Equilibrium: of the 175 tests across all loci by all sites, only eight were significant after bonferroni correction (Table S2). For just one of these significant departures from HWE did the *F*_IS_ value represent an excess of heterozygotes (at locus *Orcfra13* for site NEa at Casey; Table S2). The remaining seven were heterozygote deficits at locus *Orcfra4* (Table S2) and all of these occurred at Davis sites. Heterozygote deficits can result from the presence of null alleles; indeed at *Orcfra4* we found evidence of null alleles for all nine sites at Davis, and for three of the 16 sites at Casey. We subsequently adjusted allele frequencies at *Orcfra4* to account for the presence of null alleles (using the Oosterhout correction algorithm in Microchecker), but this made no difference to the significance of genetic differentiation or isolation by distance determined under either the IAM or SSM, therefore results for the raw data alone are presented for simplicity. Evidence of null alleles was also detected at *Orcfra5* (in five sites), and *Orcfra6* (in three sites). Adjusting allele frequencies at these loci was considered unnecessary because the null alleles were only detected in a small proportion of the total 25 sites.

There was no evidence of cryptic species within the samples of *O. franklini* from Casey and Davis. Although PCoA explained 67% of the variation in the multilocus genetic data within the first three co-ordinates, it indicated just a single genetic group within the sample (Fig. S1).

### Population differentiation

Genetic differentiation among all sites was highly significant, regardless of the mutational model assumed (*F*_ST_ = 0.086, p<0.001; *R*_ST_ = 0.139, p<0.001; 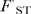 = 0.162; Table 3). Hierarchical AMOVA revealed that the majority of this genetic differentiation occurred between Casey and Davis (69%), with significant differentiation also occurring among locations within each region (2%), but not among sites within each location (Table 4). A considerable amount of the variation (29%) was also due to differences among individuals within each site (Table 4). This hierarchical structure was also reflected in pairwise differentiation estimates: *F*_ST_ between Casey sites and Davis sites ranged from 0.120 to 0.199, whereas pairwise *F*_ST_ estimates among all sites within Casey and Davis ranged from 0 to 0.031 (Table S3). Pairwise *R*_ST_ estimates were consistently higher than *F*_ST_ values, but showed the same pattern; ranging from 0.063 to 0.486 between Casey and Davis, from 0.000 to 0.119 within Casey, and from 0.000 to 0.129 within Davis (Table S3). The estimate of migration (*N_e_m*) occurring between Casey and Davis was 1.4 (both before and after the removal of loci under selection; see below).

**Table 3:** Estimates of genetic differentiation (*F*_ST_, *R*_ST_ and *F*′_ST_) among all sites for *Orchomenella franklini*.

	*F*_ST_	*R*_ST_	*F*′_ST_
*Orcfra3*	0.091	0.177	0.254
*Orcfra4*	0.206	0.234	0.205
*Orcfra5*	0.012	0	0.013
*Orcfra6*	0.056	0.027	0.146
*Orcfra12*	0.049	0.147	0.128
*Orcfra13*	0.089	0.133	0.159
*Orcfra26*	0.154	0.161	0.190
Overall:	0.086***	0.139***	0.162
	(0.085***)		

Estimates of genetic differentiation are given for each locus and over all loci. The overall estimate of *F*_ST_ excluding loci potentially under selection (*Orcfra4*, *Orcfra5* and *Orcfra12*) is provided in parentheses. Negative values have been converted to zero. Significance of differentiation is indicated as ***p<0.001.

**Table 4:** The partitioning of genetic variation in *Orchomenella franklini* at each spatial level as indicated by hierarchical AMOVA.

	Over all loci			Excluding potentially selected loci		
	F-statistic	var. component	% variance	F-statistic	var. component	% variance
Among regions	0.156***	0.640	68.7	0.152***	0.393	86.4
Among locations within regions	0.005**	0.019	2.0	0.006**	0.014	3.0
Among sites within locations	0	0	0	0	0	0
Within sites	0.079	0.273	29.3	0.002	0.048	10.6

Results of the analysis excluding loci potentially under selection (*Orcfra4*, *Orcfra5* and *Orcfra12*) are also provided. Negative values have been converted to zero. Significance is indicated as *p<0.05, **p<0.01, ***p<0.001.

Locus *Orcfra4* produced the highest overall *F*_ST_ values (Table 3), and we found evidence that this locus was under directional selection (as indicated by 99% confidence intervals). We also detected balancing selection at the two loci which produced the lowest overall *F*_ST_ estimates: *Orcfra5* and *Orcfra12* (Table 3). We subsequently removed these three loci from the analyses, but the results remained unchanged (Tables 3 and 4). *F*_ST_ estimates for the differentiation of locations within regions were not particularly high for *Orcfra4*, nor were they particularly low for *Orcra5* and *Orcfra12*. Indeed, when we tested loci for evidence of selection within the Casey and Davis datasets independently, all loci were found to be neutral. This suggests that selection associated with *Orcfra4*, *Orcfra5* and *Orcfra12* is occurring at the regional scale (i.e. between Casey and Davis) but not on a smaller scale (i.e. between populations within each of the regions).

**Figure 2:**
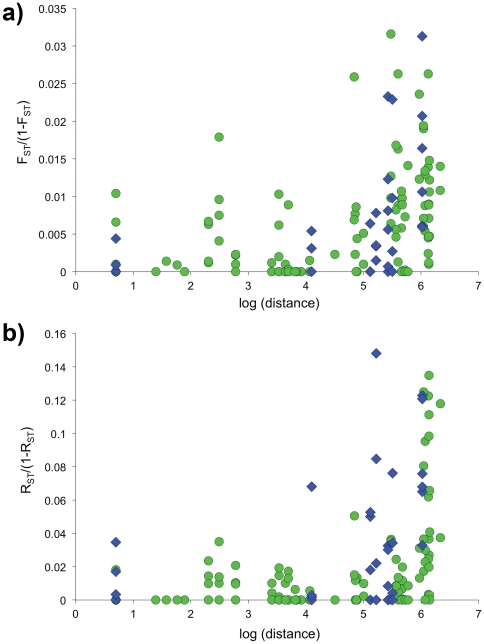
The relationship between geographic distance and a) linearised *F*_ST_, and b) linearised *R*_ST_, for *Orchomenella franklini*. In both cases, genetic isolation by distance was significant (see Results). Green circles represent data from Casey; blue diamonds represent data from Davis.

We found evidence of isolation by distance indicative of stepping-stone dispersal among sites within both the Casey and Davis regions. Mantel tests indicated a significant correlation between genetic and geographic distance under both the IAM (Casey: p = 0.002; Davis: p = 0.000; Fig. 2A), and the SMM (Casey: p = 0.003; Davis: p = 0.019; Fig. 2B).

## Discussion

This study has revealed considerable genetic diversity and population differentiation in the ubiquitous Antarctic benthic invertebrate, *Orchomenella franklini*. Genetic differentiation was most pronounced across 1000 s of km between Casey and Davis, indicating that populations in these two regions are effectively isolated. At local scales, genetic differentiation was consistent with a stepping-stone model of dispersal and we conclude that individuals maintain gene flow over hundreds of metres, but that dispersal across larger distances occurs rarely. In addition, we found evidence of differential selection occurring between the Casey and Davis populations and suggest this represents localised adaptation in *O. franklini*. The lack of gene flow among populations and evidence of selection provide an insight into processes of speciation in Antarctic brooders, and should be considered in future management initiatives for the Antarctic benthos.

### Contrasting levels of genetic diversity in *O. franklini* populations

While microsatellite variation in all *O. franklini* populations was considerable, genetic diversity was significantly lower at Casey than at Davis. Contrasting levels of genetic diversity among populations of high latitude species are often suggested to reflect signatures of historical glaciation [31], [46], [96], however this is unlikely to explain our data, as microsatellite variation should reflect more recent demographic processes [97]. One potential explanation is differences in the spatial heterogeneity of Casey and Davis: it is generally accepted that greater environmental heterogeneity will maintain a higher level of genetic variation within species (reviewed in [98]). While there is no published comparison of Casey and Davis environments, studies have generally emphasised the high heterogeneity of Davis benthic habitats, which include fjords and fjord mouths, open wave-exposed coast and significant quantities of wind blown sediment resulting from large ice-free areas [99], [100]. Preliminary measurements of sediment properties also indicate greater heterogeneity at Davis, with a range in mean sediment grain size range among Davis locations of approximately 516 µm and a range among Casey locations of approximately 222 µm. Similarly, the total organic carbon content of sediments ranges up to 15% at Davis, and only up to 10% at Casey (the authors, unpublished data). Different levels of iceberg scouring (see [47]) and interspecific competition (see [48]) have also been proposed to explain contrasting genetic diversity among Antarctic benthic invertebrate populations, providing further plausible explanations for the observed differences between Casey and Davis.

There was some evidence of an effect of local anthropogenic pollution on genetic diversity. Although allelic richness and heterozygosity measures appeared unaffected, the number of private alleles per population was lower in polluted locations. Private alleles are an important measure of genetic diversity [101], and a reduction in private alleles may occur through many mechanisms including population bottlenecks, selection against sensitive genotypes or even depressed mutation rates due to contaminants [26]. While elucidating the contribution of these processes to reduced genetic diversity in polluted *O. franklini* populations is beyond the scope of this study, the result indicates that further examination of the genetic effects of anthropogenic pollution on Antarctic benthos will be important. Interestingly, significantly lower genetic diversity has also been observed for contaminated populations of the amphipod *Orchomenella pinguis* from the Arctic [102], suggesting that amphipods may be useful bioindicators of anthropogenic induced genetic change in polar regions.

### Restricted gene flow in *O. franklini*

Genetic differentiation among populations of *O. franklini* was greatest between the two major geographical regions Casey and Davis, which explained 69% of all microsatellite variation observed. Significant *F*_ST_ of 0.16, pairwise *F*_ST_ values of up to 0.2, and *R*_ST_ values up to nearly 0.5 indicate that the two regions are effectively isolated. Importantly, this significant genetic differentiation was still evident after removal of loci under selection, confirming that genetic drift due to restricted gene flow is important in driving this strong genetic subdivision between Casey and Davis. Estimated *N_e_m* of 1.4 provides further evidence that there is insufficient exchange of individuals between these regions to prevent them from diverging on independent evolutionary trajectories [4], [103].

Gene flow in *O. franklini* is also limited across relatively small spatial scales. Although there was no significant differentiation revealed between replicate sites within locations, indicating that animals are panmictic over 100 s of metres, we did find genetic differentiation among locations within regions (i.e. across distances of 1–30 km). There was a clear pattern of isolation by distance within both Casey and Davis, indicating that migration occurs primarily between adjacent populations [104]. This is one of the first reports of population differentiation over such a small distance for an Antarctic benthic invertebrate. Limited gene flow over these scales is consistent with the brooding development in *O. franklini*, which predicts highly restricted capacity for dispersal. Indeed, similar findings have been reported for brooding taxa from temperate and tropical regions (e.g. [105]–[108]). Whilst some studies of brooding Antarctic invertebrates have also revealed strong intraspecific structure [46], [49], other Antarctic brooders have shown evidence of gene flow over remarkably large distances, purported to reflect passive dispersal via the Antarctic Circumpolar Current [48], [109]. Clearly, such a mode of dispersal does not occur in *O. franklini*, despite its wide distribution around the Antarctic coast [110], as verified in this study by the absence of any evidence for cryptic species. Similar to conclusions drawn by [49] for a circum-Antarctic brooding pycnogonid, we suggest that *O. franklini* has achieved its widespread distribution through historical colonisation, but that contemporary gene flow over large distances is severely limited. Such restricted gene flow is likely to promote allopatric speciation between populations and therefore supports the notion that limited dispersal has contributed to the high species diversity observed in Antarctic amphipods [50] and brooding taxa in general [111].

### Evidence of local adaptation

We detected directional selection acting between Casey and Davis populations of *O. franklini* at locus *Orcfra4*, providing further evidence that these two regions are isolated and evolving independently. Although microsatellites are considered a neutral marker, they have increasingly been shown to reflect selection by genetic hitch-hiking [93], [112], [113]. Rather than rendering them uninformative, this provides valuable, biologically-relevant information on population structure [114], [115]. Selection did not appear to occur between locations or sites within each region, suggesting there exists a large-scale selection pressure, to which populations across entire regions are differentially adapted. This selection provides a likely explanation for the significant inbreeding observed at *Orcfra4* for most Davis populations, yet none from Casey. Additionally, loci *Orcfra5* and *Orcfra12* showed evidence of balancing selection, indicating that homogenizing selection pressures also act across both of the regions.

Localised adaptation of Antarctic benthic populations has barely been researched to date, as the stability of the environment has long fostered the view of a relatively homogenous fauna [116]–[119]. Whilst this theory has since been dispelled by observations of distinctly heterogenous species assemblages [120], [121], little genetic research has addressed the issue. Locally adapted populations may reduce the potential for a species to respond cohesively to broad-scale environmental change, as advantageous alleles will not have the opportunity to become widespread [4], [5]. Rather, local adaptation is likely to facilitate speciation, as populations subject to differential selection pressures become more genetically isolated over time [122], [123]. For *O. franklini*, the potential for speciation between Casey and Davis populations will ultimately be determined by the interplay of both directional and balancing selective forces, along with continued genetic drift in the face of restricted gene flow.

### Implications for conservation and future research

The geographical isolation of *O. franklini* populations has important implications for the future design of Antarctic MPAs. To maintain connectivity in this species and replenish any diminished populations outside reserve boundaries, a very close spacing of protected areas would be required. Of course, final management designs must incorporate such information from a wide variety of taxa, nevertheless, the high prevalence of brooding in Antarctic benthic species [59], [124] suggests that maintaining connectivity between reserves will emerge as a key design challenge. Of further importance to Antarctic benthic management is the different levels of genetic diversity observed within *O. franklini* (e.g. between Casey and Davis populations). Conserving genetic diversity within species is crucial as it provides the raw material for adaptation to changing conditions, hence facilitating long-term persistence [8], [125]. Thus, if management efforts inadvertently protect populations with lower genetic diversity, as has already been shown to occur in one established marine reserve (see [9]), the evolutionary potential of species may be compromised. Our study also provided preliminary evidence of a loss of genetic diversity in polluted populations, which may further increase their susceptibility to any ongoing stressors [126], [127]. Such indications of anthropogenic induced genetic change require further attention in the Antarctic, where pollutants are highly localised [38], yet their effects on marine fauna are largely unknown [128].

Clearly, intraspecific genetic structure is a field that warrants increased research in the Antarctic benthos. To date this has been hampered by the logistical difficulties of sampling such an extreme environment [129], as well as by the common discovery of cryptic species, which drastically lowers intraspecific sample size. Our results highlight that future research should address intraspecific gene flow over several spatial scales, as mechanisms acting over one scale may not be apparent over another. Despite Antarctica's suite of remarkably stable environmental features and long-held views of a homogenous fauna, our study suggests that populations may be adapted to local selection pressures within the Antarctic benthic environment, and this may help explain the high rates of speciation in amphipods and other Antarctic brooders. The continued use of microsatellites and other highly variable molecular markers should further illuminate such microevolutionary patterns in the Antarctic benthos [32], although increased research on the underlying ecology of species will help interpret the patterns revealed, in particular the processes driving local adaptation. Ultimately, this will improve our understanding of Antarctic benthic species responses to environmental change, and how best to manage this unique environment.

## Supporting Information

Figure S1 **Results of Principal Coordinate Analysis on multilocus genotypes of** ***Orchomenella franklini*** **.** Over 50% of genetic variation is explained within the first two coordinates, however, there is no single group sufficiently discrete to indicate cryptic species in the dataset.
(TIF)

Table S1 **Regions, locations, and sites sampled for** ***Orchomenella franklini*** **, with corresponding sample size (** ***n*** **).** Locations (and respective sites) in bold have been classified as polluted.
(DOC)

Table S2 **Inbreeding coefficients by locus (** ***F*** **_IS_) and the number of private alleles (** ***P_A_*** **) for each population of** ***Orchomenella franklini*** **.** Asterisks indicate significant departures from Hardy-Weinberg Equilibrium (p<0.05) after Bonferroni correction. Dashes indicate that a locus was monomorphic, hence *F*_IS_ could not be estimated. Polluted sites are in bold.
(DOC)

Table S3 **Matrix of pairwise differentiation estimates for all** ***Orchomenella franklini*** **populations sampled at Casey and Davis.** *F*_ST_ below diagonal; *R*_ST_ above diagonal. Estimates of differentiation *between* Casey and Davis populations are italicised. Negative values have been converted to zero. Polluted sites are in bold.
(DOC)
