# Supplementary figures and images for: Genetic Population Structure in the Antarctic Benthos: Insights from the Widespread Amphipod, Orchomenella franklini
Source: PLoS One. 2012 Mar 27;7(3):e34363. doi: 10.1371/journal.pone.0034363 (PMC3313966; doi:10.1371/journal.pone.0034363)

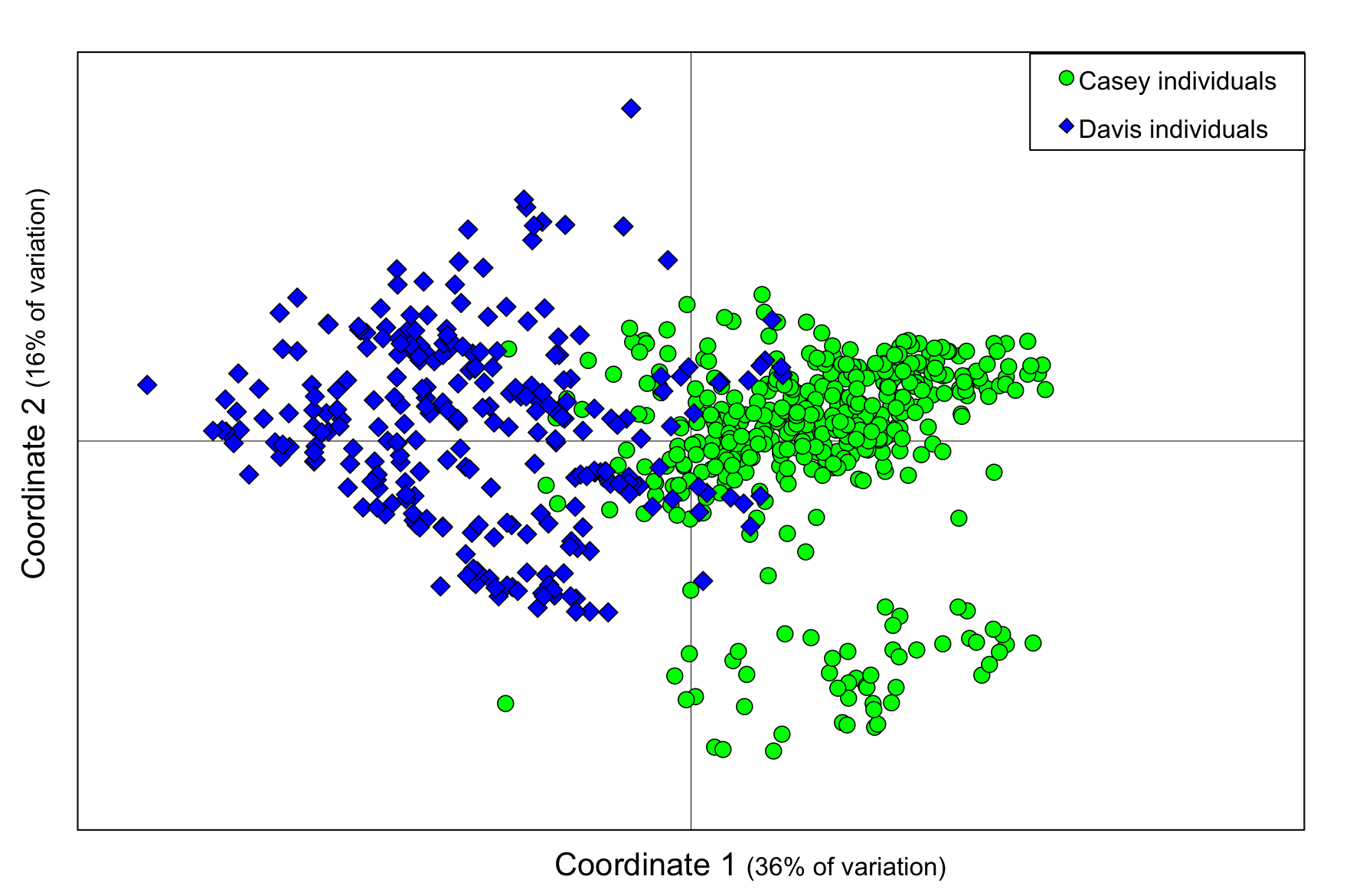

Supplement: Figure S1 — Results of Principal Coordinate Analysis on multilocus genotypes of Orchomenella franklini . Over 50% of genetic variation is explained within the first two coordinates, however, there is no single group sufficiently discrete to indicate cryptic species in the dataset. (TIF) [file pone.0034363.s001.tif]
