# Supplementary material for: Genetic Population Structure in the Antarctic Benthos: Insights from the Widespread Amphipod, Orchomenella franklini
Source: PLoS One. 2012 Mar 27;7(3):e34363. doi: 10.1371/journal.pone.0034363 (PMC3313966; doi:10.1371/journal.pone.0034363)
Supplement: Table S1 — Regions, locations, and sites sampled for Orchomenella franklini , with corresponding sample size ( n ). Locations (and respective sites) in bold have been classified as polluted. (DOC) [file pone.0034363.s002.doc]

**Table S1: Regions, locations, and sites sampled for *O. franklini*, with corresponding sample size (*n*).**

| **Region** | **Location (code)** | **Site** | ***n*** |
| --- | --- | --- | --- |
| Casey | Honkala (HO) | HO | 30 |
|  | McGrady (MG) | MGa | 19 |
|  |  | MGb | 32 |
|  | Peterson (PE) | PEa | 30 |
|  |  | PEb | 31 |
|  | Sparkes (SP) | SPa | 26 |
|  |  | SPb | 30 |
|  | **Brown (BB)** | **BBa** | 30 |
|  |  | **BBb** | 30 |
|  |  | **BBc** | 30 |
|  |  | **BBd** | 14 |
|  | **Newcombe (NE)** | **NEa** | 30 |
|  |  | **NEb** | 30 |
|  | **Shannon (SH)** | **SHa** | 30 |
|  |  | **SHb** | 30 |
|  | **Wilkes (WK)** | **WK** | 26 |
|  | *Total for Casey:* | | *448* |
| Davis | Old Wallow (OW) | OWa | 30 |
|  |  | OWb | 30 |
|  | Sorsdal (SD) | SDa | 30 |
|  |  | SDb | 30 |
|  |  | SDc | 30 |
|  | Zappet (ZP) | ZPa | 30 |
|  |  | ZPb | 30 |
|  | **Wharf (WH)** | **WHa** | 30 |
|  |  | **WHb** | 30 |
|  | *Total for Davis:* | | *270* |

Locations (and respective sites) in bold have been classified as polluted.
