# Supplementary material for: Genetic Population Structure in the Antarctic Benthos: Insights from the Widespread Amphipod, Orchomenella franklini
Source: PLoS One. 2012 Mar 27;7(3):e34363. doi: 10.1371/journal.pone.0034363 (PMC3313966; doi:10.1371/journal.pone.0034363)
Supplement: Table S2 — Inbreeding coefficients by locus ( F IS) and the number of private alleles ( PA ) for each population of Orchomenella franklini . Asterisks indicate significant departures from Hardy-Weinberg Equilibrium (p<0.05) after Bonferroni correction. Dashes indicate that a locus was monomorphic, hence F IS could not be estimated. Polluted sites are in bold. (DOC) [file pone.0034363.s003.doc]

***Table S2: Inbreeding coefficients by locus (F_IS_) and the number of private alleles (P_A_) for each population of*** O. franklini

|  |  |  | *F*_IS_ | | | | | | |
| --- | --- | --- | --- | --- | --- | --- | --- | --- | --- |
|  |  | *P_A_* | *Orcfra3* | *Orcfra4* | *Orcfra5* | *Orcfra6* | *Orcfra12* | *Orcfra13* | *Orcfra26* |
| Casey | HO | 2 | 0.141 | 0.791 | 0.659 | 0.072 | 0.117 | -0.394 | -0.047 |
|  | MGa | 0 | 0.004 | 0 | -0.015 | -0.042 | -0.011 | 0.129 | -0.029 |
|  | MGb | 0 | 0.043 | -0.034 | -0.018 | 0.187 | 0.008 | 0.021 | -0.008 |
|  | PEa | 1 | -0.216 | -0.010 | -0.020 | 0.037 | 0.018 | 0.056 | --- |
|  | PEb | 0 | 0.019 | -0.008 | -0.018 | 0.046 | -0.054 | 0.142 | --- |
|  | SPa | 0 | -0.096 | -0.042 | 0.653 | 0.057 | 0.147 | 0.031 | 0 |
|  | SPb | 1 | 0.162 | 0.458 | 0 | 0.131 | 0.014 | 0.010 | -0.009 |
|  | **BBa** | 0 | 0.162 | -0.018 | 0.477 | -0.091 | 0.103 | -0.037 | --- |
|  | **BBb** | 0 | 0.105 | -0.020 | -0.020 | -0.013 | 0.024 | -0.092 | --- |
|  | **BBc** | 0 | -0.014 | -0.009 | -0.020 | 0.035 | -0.073 | 0.172 | --- |
|  | **BBd** | 0 | -0.044 | 0 | --- | 0.142 | 0.265 | 0.524 | 0 |
|  | **NEa** | 0 | 0.077 | -0.036 | -0.059 | -0.153 | 0.158 | -0.607* | 0 |
|  | **NEb** | 0 | 0.042 | 0.728 | --- | -0.001 | 0.051 | 0.113 | 0 |
|  | **SHa** | 1 | -0.074 | -0.009 | --- | -0.071 | -0.023 | -0.040 | 0 |
|  | **SHb** | 0 | 0.134 | 0.220 | 0.786 | 0.114 | 0.122 | 0.161 | --- |
|  | **WK** | 0 | -0.015 | -0.029 | 1.000 | -0.080 | 0.068 | -0.308 | 0 |
| Davis | OWa | 3 | 0.132 | 0.604* | 0.419 | -0.106 | -0.072 | -0.020 | -0.009 |
|  | OWb | 0 | 0.081 | 0.827* | 0.292 | 0.173 | -0.020 | -0.072 | 0.192 |
|  | SDa | 1 | -0.085 | 0.587* | 0.454 | 0.203 | 0.044 | 0.251 | 0.163 |
|  | SDb | 2 | -0.254 | 0.679* | 0.190 | -0.123 | 0.091 | 0.095 | 0.139 |
|  | SDc | 3 | -0.043 | 0.594* | 0.377 | 0.232 | -0.037 | -0.127 | -0.148 |
|  | ZPa | 1 | 0.158 | 0.421 | 0.794 | 0.009 | 0.043 | -0.210 | -0.028 |
|  | ZPb | 1 | 0.172 | 0.893* | -0.018 | 0.012 | -0.023 | -0.040 | -0.182 |
|  | **WHa** | 0 | 0.029 | 0.425 | -0.049 | 0.423 | -0.025 | -0.095 | 0.348 |
|  | **WHb** | 0 | 0.106 | 0.736* | 0.663 | 0.023 | -0.059 | -0.128 | -0.226 |

Asterisks indicate significant departures from Hardy-Weinberg Equilibrium (p<0.05) after Bonferroni correction. Dashes indicate that a locus was monomorphic, hence *F*_IS_ could not be estimated. Polluted sites are in bold.
