# Supplementary material for: Genetic Population Structure in the Antarctic Benthos: Insights from the Widespread Amphipod, Orchomenella franklini
Source: PLoS One. 2012 Mar 27;7(3):e34363. doi: 10.1371/journal.pone.0034363 (PMC3313966; doi:10.1371/journal.pone.0034363)
Supplement: Table S3 — Matrix of pairwise differentiation estimates for all Orchomenella franklini populations sampled at Casey and Davis. F ST below diagonal; R ST above diagonal. Estimates of differentiation between Casey and Davis populations are italicised. Negative values have been converted to zero. Polluted sites are in bold. (DOC) [file pone.0034363.s004.doc]

**Table S3: Matrix of pairwise differentiation estimates for all *O. franklini* populations sampled at Casey and Davis**.

|  | HO | MGa | MGb | PEa | PEb | SPa | SPb | **BBa** | **BBb** | **BBc** | **BBd** | **NEa** | **NEb** | **SHa** | **SHb** | **WK** | OWa | OWb | SDa | SDb | SDc | ZPa | ZPb | **WHa** | **WHb** |
| --- | --- | --- | --- | --- | --- | --- | --- | --- | --- | --- | --- | --- | --- | --- | --- | --- | --- | --- | --- | --- | --- | --- | --- | --- | --- |
| HO | - | 0.002 | 0.010 | 0.106 | 0.036 | 0.013 | 0.030 | 0.000 | 0.013 | 0.000 | 0.012 | 0.048 | 0.015 | 0.000 | 0.000 | 0.000 | *0.202* | *0.309* | *0.138* | *0.129* | *0.161* | *0.362* | *0.321* | *0.216* | *0.124* |
| MGa | 0.001 | - | 0.000 | 0.062 | 0.003 | 0.000 | 0.000 | 0.000 | 0.000 | 0.000 | 0.004 | 0.000 | 0.000 | 0.000 | 0.000 | 0.000 | *0.170* | *0.287* | *0.095* | *0.087* | *0.122* | *0.350* | *0.309* | *0.197* | *0.086* |
| MGb | 0.005 | 0.000 | - | 0.039 | 0.000 | 0.000 | 0.000 | 0.002 | 0.015 | 0.000 | 0.010 | 0.019 | 0.000 | 0.000 | 0.006 | 0.005 | *0.223* | *0.340* | *0.145* | *0.134* | *0.174* | *0.400* | *0.357* | *0.253* | *0.134* |
| PEa | 0.011 | 0.015 | 0.012 | - | 0.000 | 0.035 | 0.034 | 0.100 | 0.119 | 0.058 | 0.109 | 0.087 | 0.029 | 0.074 | 0.111 | 0.090 | *0.314* | *0.435* | *0.219* | *0.203* | *0.251* | *0.486* | *0.452* | *0.345* | *0.207* |
| PEb | 0.014 | 0.010 | 0.005 | 0.007 | - | 0.000 | 0.000 | 0.026 | 0.035 | 0.003 | 0.024 | 0.022 | 0.000 | 0.017 | 0.035 | 0.019 | *0.228* | *0.341* | *0.146* | *0.137* | *0.180* | *0.399* | *0.355* | *0.264* | *0.137* |
| SPa | 0.012 | 0.000 | 0.000 | 0.006 | 0.010 | - | 0.000 | 0.001 | 0.007 | 0.000 | 0.001 | 0.007 | 0.000 | 0.000 | 0.009 | 0.000 | *0.202* | *0.321* | *0.120* | *0.111* | *0.152* | *0.380* | *0.337* | *0.233* | *0.112* |
| SPb | 0.023 | 0.007 | 0.000 | 0.031 | 0.013 | 0.001 | - | 0.012 | 0.019 | 0.000 | 0.006 | 0.013 | 0.005 | 0.008 | 0.024 | 0.009 | *0.231* | *0.358* | *0.141* | *0.129* | *0.173* | *0.418* | *0.377* | *0.264* | *0.133* |
| **BBa** | 0.000 | 0.000 | 0.000 | 0.009 | 0.001 | 0.000 | 0.010 | - | 0.000 | 0.000 | 0.000 | 0.010 | 0.009 | 0.000 | 0.000 | 0.000 | *0.173* | *0.279* | *0.105* | *0.100* | *0.132* | *0.340* | *0.293* | *0.198* | *0.098* |
| **BBb** | 0.004 | 0.000 | 0.002 | 0.007 | 0.001 | 0.000 | 0.009 | 0.000 | - | 0.000 | 0.000 | 0.000 | 0.020 | 0.000 | 0.000 | 0.000 | *0.153* | *0.263* | *0.083* | *0.080* | *0.112* | *0.330* | *0.278* | *0.185* | *0.078* |
| **BBc** | 0.002 | 0.000 | 0.000 | 0.014 | 0.004 | 0.006 | 0.011 | 0.000 | 0.001 | - | 0.000 | 0.010 | 0.000 | 0.000 | 0.000 | 0.000 | *0.198* | *0.309* | *0.122* | *0.115* | *0.152* | *0.368* | *0.322* | *0.226* | *0.114* |
| **BBd** | 0.008 | 0.001 | 0.000 | 0.026 | 0.002 | 0.010 | 0.000 | 0.000 | 0.001 | 0.000 | - | 0.014 | 0.034 | 0.010 | 0.000 | 0.000 | *0.143* | *0.252* | *0.072* | *0.063* | *0.097* | *0.309* | *0.270* | *0.164* | *0.072* |
| **NEa** | 0.025 | 0.000 | 0.006 | 0.013 | 0.009 | 0.001 | 0.016 | 0.002 | 0.001 | 0.007 | 0.010 | - | 0.018 | 0.014 | 0.023 | 0.017 | *0.152* | *0.267* | *0.074* | *0.071* | *0.106* | *0.341* | *0.286* | *0.195* | *0.070* |
| **NEb** | 0.007 | 0.000 | 0.010 | 0.013 | 0.005 | 0.009 | 0.026 | 0.000 | 0.002 | 0.004 | 0.018 | 0.010 | - | 0.000 | 0.010 | 0.013 | *0.214* | *0.326* | *0.139* | *0.132* | *0.169* | *0.386* | *0.343* | *0.244* | *0.126* |
| **SHa** | 0.009 | 0.000 | 0.000 | 0.019 | 0.006 | 0.008 | 0.017 | 0.000 | 0.009 | 0.000 | 0.001 | 0.006 | 0.001 | - | 0.000 | 0.000 | *0.184* | *0.291* | *0.118* | *0.112* | *0.144* | *0.351* | *0.307* | *0.211* | *0.108* |
| **SHb** | 0.000 | 0.000 | 0.000 | 0.019 | 0.007 | 0.005 | 0.006 | 0.000 | 0.000 | 0.000 | 0.000 | 0.007 | 0.001 | 0.000 | - | 0.000 | *0.179* | *0.284* | *0.115* | *0.110* | *0.140* | *0.343* | *0.298* | *0.201* | *0.106* |
| **WK** | 0.002 | 0.000 | 0.002 | 0.009 | 0.005 | 0.000 | 0.014 | 0.000 | 0.000 | 0.000 | 0.000 | 0.000 | 0.000 | 0.000 | 0.000 | - | *0.176* | *0.285* | *0.105* | *0.098* | *0.133* | *0.343* | *0.298* | *0.199* | *0.095* |
| OWa | *0.151* | *0.153* | *0.166* | *0.164* | *0.148* | *0.169* | *0.165* | *0.152* | *0.143* | *0.168* | *0.144* | *0.137* | *0.138* | *0.167* | *0.147* | *0.141* | - | 0.017 | 0.000 | 0.000 | 0.000 | 0.071 | 0.033 | 0.002 | 0.003 |
| OWb | *0.136* | *0.140* | *0.156* | *0.144* | *0.129* | *0.153* | *0.160* | *0.137* | *0.124* | *0.155* | *0.134* | *0.121* | *0.120* | *0.155* | *0.140* | *0.125* | 0.000 | - | 0.048 | 0.050 | 0.018 | 0.004 | 0.000 | 0.000 | 0.064 |
| SDa | *0.150* | *0.155* | *0.172* | *0.152* | *0.141* | *0.166* | *0.171* | *0.150* | *0.136* | *0.169* | *0.154* | *0.131* | *0.132* | *0.170* | *0.152* | *0.141* | 0.000 | 0.000 | - | 0.000 | 0.000 | 0.109 | 0.061 | 0.032 | 0.000 |
| SDb | *0.147* | *0.150* | *0.163* | *0.150* | *0.138* | *0.160* | *0.163* | *0.146* | *0.134* | *0.163* | *0.144* | *0.127* | *0.131* | *0.162* | *0.146* | *0.134* | 0.000 | 0.000 | 0.000 | - | 0.000 | 0.108 | 0.064 | 0.029 | 0.000 |
| SDc | *0.181* | *0.188* | *0.196* | *0.185* | *0.173* | *0.197* | *0.196* | *0.180* | *0.169* | *0.199* | *0.182* | *0.162* | *0.165* | *0.198* | *0.179* | *0.173* | 0.000 | 0.006 | 0.000 | 0.000 | - | 0.070 | 0.032 | 0.008 | 0.000 |
| ZPa | *0.145* | *0.155* | *0.169* | *0.153* | *0.143* | *0.164* | *0.167* | *0.154* | *0.136* | *0.170* | *0.153* | *0.136* | *0.132* | *0.169* | *0.148* | *0.134* | 0.010 | 0.003 | 0.006 | 0.006 | 0.020 | - | 0.003 | 0.022 | 0.129 |
| ZPb | *0.141* | *0.152* | *0.163* | *0.144* | *0.129* | *0.158* | *0.169* | *0.146* | *0.126* | *0.162* | *0.143* | *0.124* | *0.127* | *0.162* | *0.149* | *0.129* | 0.022 | 0.000 | 0.011 | 0.016 | 0.030 | 0.004 | - | 0.000 | 0.078 |
| **WHa** | *0.141* | *0.146* | *0.163* | *0.154* | *0.140* | *0.164* | *0.167* | *0.151* | *0.135* | *0.166* | *0.143* | *0.134* | *0.132* | *0.163* | *0.149* | *0.136* | 0.005 | 0.000 | 0.012 | 0.008 | 0.023 | 0.008 | 0.003 | - | 0.034 |
| **WHb** | *0.150* | *0.158* | *0.176* | *0.159* | *0.149* | *0.174* | *0.182* | *0.158* | *0.145* | *0.174* | *0.158* | *0.138* | *0.136* | *0.173* | *0.158* | *0.140* | 0.003 | 0.000 | 0.000 | 0.001 | 0.006 | 0.003 | 0.002 | 0.001 | - |

*F*_ST_ below diagonal; *R*_ST_ above diagonal. Estimates of differentiation *between* Casey and Davis populations are italicized. Negative values have been converted to zero. Polluted sites are in bold.
